# Exploring the mechanism of action of succinic acid in ovarian cancer via single-cell sequencing of the tumor immune microenvironment

**DOI:** 10.3389/fonc.2025.1535504

**Published:** 2025-03-24

**Authors:** Jiao Zhao, Panpan Guo, Lili Zhao, Xiaobin Wang

**Affiliations:** ^1^ Department of Gynaecology, Cancer Hospital of Dalian University of Technology (Liaoning Cancer Institute and Hospital), Shenyang, Liaoning, China; ^2^ School of Biomedical Engineering, Faculty of Medicine, Dalian University of Technology, Dalian, China

**Keywords:** succinic acid, ovarian cancer, immune cell infiltration, single-cell RNA sequencing, SPP1

## Abstract

**Background:**

The main treatments for ovarian cancer are surgery, chemotherapy, radiotherapy, and targeted therapy. Targeted therapy is a new treatment method that has emerged in recent years and relies on specific molecular targets to treat cancer. Succinic acid is a key intermediate product in the tricarboxylic acid cycle. Research has shown that succinic acid has antioxidant properties and can alleviate oxidative stress in cells and tissues. These findings indicate the potential application of succinic acid in antioxidant therapy and the prevention of oxidative damage. This study explored the potential targets and therapeutic mechanisms of succinic acid in ovarian cancer.

**Methods:**

Using bioinformatics and single-cell sequencing technology, the hub genes related to succinic acid and ovarian cancer and the frequency and gene expression patterns of different cell types in ovarian cancer patients and normal individuals were analyzed.

**Results:**

The frequency of immune cells, including B cells, CD4^+^ cells, CD8^+^ cells, macrophages, and plasma cells, was significantly increased in ovarian cancer patients, and the frequency of other cell types, such as endothelial cells, NK cells, and pericytes/SMCs, was decreased. Further research revealed three key hub genes: SPP1, SLPI, and CD9. The expression patterns of these genes in ovarian cancer were closely related to different cell types. SPP1 was expressed mainly in macrophages, SLPI was expressed in epithelial cells, and CD9 was expressed in pericytes/SMCs and epithelial cells. SPP1, SLPI, and CD9 and their mechanisms of action may be potential targets for the treatment of ovarian cancer with succinic acid.

**Conclusions:**

This study investigated the potential therapeutic targets and mechanisms of succinic acid in ovarian cancer and the differences in immune cell infiltration and gene expression patterns, providing important insights for future tumor immunotherapy research.

## 1 Introduction

Among all female reproductive cancers, ovarian cancer (OV) is the most lethal malignancy. According to statistics from the World Health Organization, in 2020, 314,000 cases of OV and 207,300 deaths occurred, resulting in a mortality rate of 66.02% (1). The WHO estimates that by 2040, 445,700 cases and 313,600 deaths will occur, increasing the mortality rate to 70.36% (2, 3). Among women older than 40 years, OV is the second most common malignancy after breast cancer, especially in developed countries (4, 5). Early-stage OV often presents with no symptoms; thus, it is difficult to detect (6). By the time symptoms appear, the cancer usually has progressed to an advanced stage, making treatment more challenging. The onset of OV is associated with various factors, such as genetics, age, personal and family history, and reproductive history. The risk of developing OV may also increase with the presence of ovarian cysts and exposure to certain chemicals (7, 8). Early-stage OV typically shows no symptoms, but advanced-stage OV can present symptoms such as abdominal swelling or bloating, abdominal or pelvic pain, indigestion, and fatigue (9). As cancer spreads to other parts of the body, symptoms such as constipation, urinary system issues, and breathing difficulties may also arise (10). A diagnosis usually requires a combination of physical examinations, ultrasounds, CT/MRI scans, blood tests, biochemical marker tests, and biopsies. Early diagnosis and treatment significantly increase the survival rates of OV patients (4).

However, most patients are diagnosed at an advanced stage because of the nonspecific nature of early symptoms. The difficulty of treating advanced OV contributes to a higher mortality rate (11). Treatment options include surgery, radiotherapy, chemotherapy, targeted therapy, and immunotherapy. The choice of treatment depends on the type of cancer, the patient’s age and health condition, the stage of the cancer, and other factors (12). Chemotherapy, for example, treats OV by killing tumor cells, but its side effects, such as nausea, vomiting, hair loss, anemia, and mouth sores, are inevitable (13). Additionally, chemotherapy can lead to immunosuppression, increasing the risk of infections and affecting treatment outcomes (14).

In addition to traditional treatments, new targeted therapies are underway to improve the treatment and prognosis of OV (15). Due to the nonspecific symptoms, diagnosing OV is exceptionally challenging, and most patients are diagnosed at a late stage (16). Over 70% of OV patients are diagnosed only when the disease has progressed to stages III or IV, which is one of the main reasons for the high mortality rate. However, this high mortality rate is not inevitable; an early diagnosis of OV can reduce the mortality rate by 10% to 30%. Therefore, the discovery of new diagnostic biomarkers is crucial for the treatment of OV (17). Succinic acid is an organic acid that plays various important physiological roles in the body, mainly involving energy metabolism and other biochemical processes. Abnormal energy metabolism is a common characteristic of cancer cells, which require considerable energy for growth and reproduction. Under anaerobic conditions, glycolysis becomes the main energy production pathway (18). OV cells express high levels of glucose transport proteins and enzymes on their cell membranes, allowing for increased glucose intake and energy production through glycolysis (19). With sufficient oxygen, cancer cells can utilize food energy through the tricarboxylic acid (TCA) cycle. Studies have shown that the expression of TCA cycle enzymes involved in ATP synthesis pathways is higher in OV cells than in normal ovarian cells, indicating a greater reliance on oxygen and oxidation processes for ATP production (20). Succinic acid plays a key role in the TCA cycle, which is an important metabolic pathway in cells that breaks down glucose, fats, and amino acids into energy. Its role in energy production and its effects on certain metabolic diseases, such as hereditary succinic aciduria, are significant (21). Research indicates that succinic acid has antioxidant properties, helping clear free radicals from the body and alleviating oxidative stress in cells and tissues (22). These findings suggest potential applications of succinic acid in antioxidant therapy and the prevention of oxidative damage (23). Succinic acid may also impact the immune system by regulating immune responses and suppressing inflammation (24). This study explored the potential targets and treatment mechanisms of succinic acid in OV (25). Single-cell RNA sequencing (scRNA-seq) is a technology that isolates individual cells from tissues or bodily fluids and performs a high-throughput sequencing analysis of their genetic material at the transcriptome level. Traditional sequencing technologies usually analyze entire cell populations, obtaining information on average gene expression or dominant cell groups (26, 27). In contrast, scRNA-seq has significant advantages in studies of tissues with small sample sizes and complex cellular compositions, as it can describe the characteristics of individual cells at the transcriptome level, better reflecting the cellular heterogeneity of tissues (28, 29). Using bioinformatics and single-cell sequencing technology, this study analyzed the hub genes related to succinic acid and OV, as well as the frequency and gene expression patterns of different cell types in OV patients and normal individuals (30).

## 2 Materials and methods

### 2.1 Data sources and WGCNA

The gene expression matrix utilized in this study was primarily sourced from publicly available datasets, including standardized RNA-seq data from The Cancer Genome Atlas (TCGA) and Genotype-Tissue Expression (GTEx) ovarian cancer (OV) cohorts, which were integrated from the UCSC Xena database. A total of 415 samples, encompassing both tumor and normal tissue specimens, were included in the analysis. Following gene name conversion, the intersections of 1542 RNA-binding protein-encoding gene names obtained from the literature and those downloaded from UCSC were determined, yielding a final matrix of 1479 RNA-binding protein sample gene expression profiles. Weighted Gene Co-expression Network Analysis (WGCNA) was employed for gene coexpression analysis, with an R square cut-off parameter set at 0.85 to identify genes with high correlation.

### 2.2 Differential expression analysis in OV

The gene expression matrix, which included 429 TCGA OV samples and 88 normal samples, was analyzed using the limma R package to identify differentially expressed genes (DEGs) in the OV matrix. A chromosome position analysis of DEGs in OV was subsequently conducted through an online website (http://gepia2.cancer-pku.cn/), and relevant genes were searched using the keyword “succinic acid” in the GEO dataset (https://www.genecards.org, GeneCards-SA). A Venn diagram was created on the Hiplot website to identify SA-DEGs. For further analysis of these genes, GO and KEGG enrichment analyses were performed using the DAVID database (https://david.ncifcrf.gov). Finally, the expression matrix of 62 genes was extracted from the OV matrix, and a Pearson correlation coefficient plot was generated to assess the correlations among SA-related DEGs in OV.

### 2.3 Hub gene screening and validation

The expression levels of the 62 significantly DEGs were combined with clinical information for the survival analysis. In this experiment, the survival and survminer packages in R were used to construct single-gene survival curves. Three genes (SPP1, SLPI, and CD9) were significantly associated with the survival time of OV patients (P=0.049) based on the screening criterion of P<0.05 and were used for prognostic modelling. After stratifying OV patients using the ssGSEA algorithm, the expression of SPP1, SLPI, and CD9 was validated for the survival analysis using the Kaplan-Meier plotter database with the GSE40595 dataset from the GEO database.

### 2.4 Pancancer analysis and predictive model construction

The hub genes were subjected to a single-gene pancancer analysis using various immune cell infiltration algorithms. TCGA multicancer (33 types) immune infiltration score was determined using a variety of algorithms, including TIMER, TIDE, CIBERSORT, CIBERSORT-ABS, QUANTISEQ, XCELL, MCP-COUNTER, and EPIC. The performance of different diagnostic tests was evaluated by constructing receiver operating characteristic (ROC) curves and calculating the areas under the curves (AUCs). A line graph model incorporating important predictive factors from the logistic regression and Cox analyses was established to predict the prognosis of OV patients.

### 2.5 scRNA-seq analysis

We utilized the GSE184880 dataset to conduct an in-depth analysis of the cellular composition of OV patients and normal individuals using single-cell RNA sequencing technology. The “Seurat 5.0”, “patchwork”, “tidyverse”, and “harmony” packages were used to analyze the hub genes associated with succinic acid and OV, as well as the frequencies of different cell types and their gene expression patterns in OV patients and normal individuals.

### 2.6 Western blot analysis of proteins

Western blot analysis was widely applied to determine the expression levels of genes at the protein level in this study. Protein samples (20 μg per lane) were separated by sodium dodecyl sulfate-polyacrylamide gel electrophoresis (SDS-PAGE) with a separation gel concentration of 12% and a stacking gel concentration of 5% and then transferred onto polyvinylidene fluoride (PVDF) membranes. The PVDF membranes were subsequently blocked with a blocking solution (a 5% nonfat milk mixture prepared in a 1× TBS solution containing 0.05% Tween-20) for 2 hours, followed by an overnight incubation with primary antibodies at 4°C. The primary antibodies used in this study were rabbit anti-SPP1, SLPI (1:1000, K005805P, Solarbio) and rabbit anti-GAPDH (1:10000, D110016, Sangon Biotech) antibodies. The membranes were then incubated with the secondary antibody horseradish peroxidase (HRP)-conjugated goat anti-rabbit IgG (dilution ratio 1:1000, A0208, Beyotime) at room temperature for 1–2 hours. After the membranes were washed three times with the TBST solution, the bands were visualized using an enhanced chemiluminescence (ECL) hypersensitive luminescent liquid kit (P1000-100, Applygen) and detected with a chemiluminescence detection system (FluorChem HD2, ProteinSimple). The optical density of each band was analyzed using ImageJ 1.53 software (W. Rasband, Research Services Branch, NIMH), and the protein level in each sample was normalized to the optical density of the GAPDH band in each experiment.

### 2.7 Immunohistochemical staining of human OV tissue sections for CD68, SPP1, and SLPI

Well-preserved OV sections from Liaoning Cancer Hospital were selected for immunohistochemical staining. Sections (2–3 μm thick) of paraffin-embedded OV tissue were deparaffinized and rehydrated via sequential immersion in xylene, isopropanol, absolute ethanol, 95% ethanol, and 75% ethanol. The tissue sections were subsequently boiled in a 0.01 M sodium citrate solution (pH=6.0) for antigen retrieval. After washes with TBS solution, the exposed tissues were blocked for 2 hours with immunofluorescence staining blocking solution (P0102, Beyotime). The sections were then incubated overnight at 4°C in a humidified chamber with rabbit anti-SPP1 and SLPI antibodies (dilution ratio 1:100, K005805P, Solarbio). After three washes with TBS solution and two washes with buffer of 5 minutes each, the primary antibody enhancer was added. The samples were incubated at room temperature for 20 minutes, followed by two washes with buffer for 5 minutes each. The HRP polymer (enzyme-labelled secondary antibody) was added, and the samples were incubated at room temperature for 30 minutes (note that the HRP polymer is light sensitive and should be stored in an opaque vial to avoid unnecessary light exposure). After three washes with buffer solution for 5 minutes each, 1–2 drops of DAB Plus Chromogen (or AEC Plus Chromogen) were added to 1 ml of DAB Plus Substrate (or AEC Plus Substrate) and mixed well. The mixture was applied to the sections and incubated for 3–15 minutes (the specific time depends on the desired staining intensity). The sections were thoroughly rinsed with tap water, counterstained, dehydrated, cleared, and placed on a coverslip.

### 2.8 Dual immunofluorescence staining for CD68, SPP1, and SLPI in human OV tissue sections

Due to the strong correlations among SPP1, SLPI, and macrophages revealed by our OV bioinformatics analysis, we conducted dual immunofluorescence staining targeting CD68 (a macrophage surface marker), SPP1, and SLPI to determine the cellular localization of SPP1 and SLPI in OV tissues. First, 2–3 μm thick paraffin-embedded OV tissue sections were deparaffinized and rehydrated by sequential immersion in xylene, isopropanol, absolute ethanol, 95% ethanol, and 75% ethanol. The tissue sections were subsequently boiled in a 0.01 M sodium citrate solution (pH=6.0) for antigen retrieval. After washes with TBS solution, the exposed tissues were blocked for 2 hours with immunofluorescence staining blocking solution (P0102, Beyotime). The sections were then incubated overnight at 4°C in a humidified chamber with rabbit anti-SPP1 and SLPI antibodies (dilution ratio 1:100, K005805P, Solarbio). After three washes with TBS, the sections were incubated with Cy3-conjugated goat anti-rabbit IgG (dilution ratio 1:400, GB21303, Servicebio) in the dark at room temperature for 2 hours. The tissue sections were subsequently washed three times with a freshly prepared TBS solution and incubated with a rabbit anti-CD68 antibody (dilution ratio of 1:200, abs120102, Absin) overnight at 4°C in a humidified chamber. After three washes with TBS solution, the tissue sections were incubated with goat anti-rabbit IgG-Alexa Fluor 488 (dilution ratio 1:400, abs20025, absin) in the dark at room temperature for 2 hours. Finally, the tissue sections were washed three times with TBS solution and stained with DAPI-containing antifade mounting medium (P0131, Beyotime) in the dark for 10 minutes. Fluorescence images of different fields were observed and captured using an inverted fluorescence microscope (Nikon).

### 2.9 Statistical analysis

1All the data were analyzed using SPSS 25.0 software (IBM Corp) and GraphPad Prism 8.0.1 software (GraphPad Software). Parametric tests (Student’s t test for two unpaired independent samples) or nonparametric Mann-Whitney U tests were used to analyze differences between groups, depending on the distribution of the variables. One-way ANOVA with the Brown–Forsythe test were used for comparisons between three or more groups. For the bioinformatics analysis in this study, correlations between different variables were obtained through Pearson’s correlation analysis. A P value <0.05 was considered to indicate statistical significance. Moreover, all the experiments in this study were repeated three times to ensure the reproducibility of the results.

## 3 Results

### 3.1 Selection of coexpressed genes

**Figure 1:**
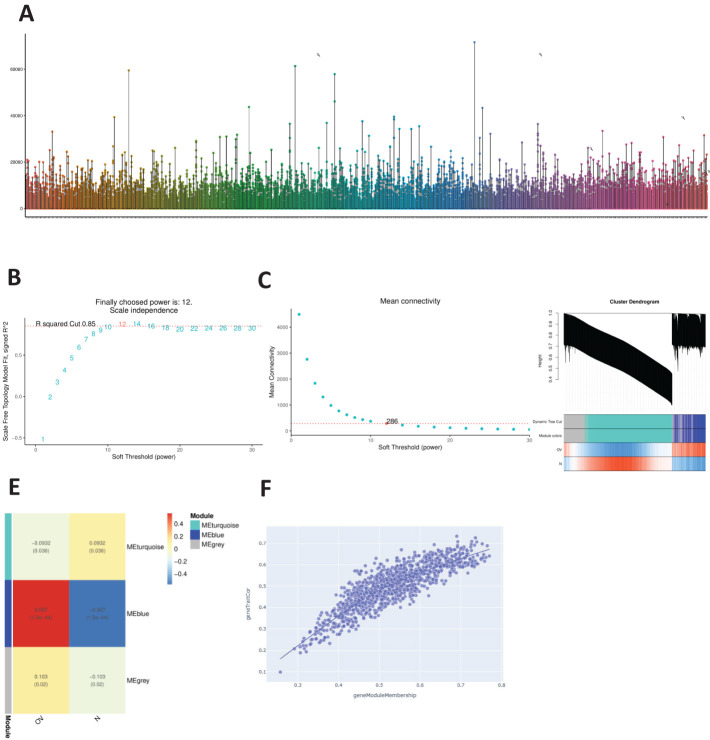
WGCNA. **(A)** Distribution of the expression profiles for all samples. **(B)** Relationships between the scale-free fit index and various soft-thresholding powers. **(C)** Relationships between average connectivity and various soft-thresholding powers. **(D)** Module-trait association graph. **(E)** Module characteristic graph. **(F)** Gene dendrogram of the blue module.

The distributions of the expression profiles of all the samples obtained via WGCNA are shown in **Figure 1A**. We chose a soft threshold of 12 (based on the scale-free topology criterion R^2^ = 0.85) to construct a scale-free network. The adjacency matrix was transformed into a TOM matrix (**Figures 1B, C**) to display the similarity between nodes by considering weighted correlations. We subsequently created module–gene trait correlation plots (**Figure 1D**) and module–feature correlation plots (**Figure 1E**) using a correlation matrix generated by Pearson’s correlation coefficients and identified six modules through average hierarchical clustering and dynamic tree cutting. The blue module strongly correlated with OV (**Figure 1F**); therefore, the intersection of this module with the DEGs in OV was further analyzed.

### 3.2 Differential expression analysis of OV

**Figure 2:**
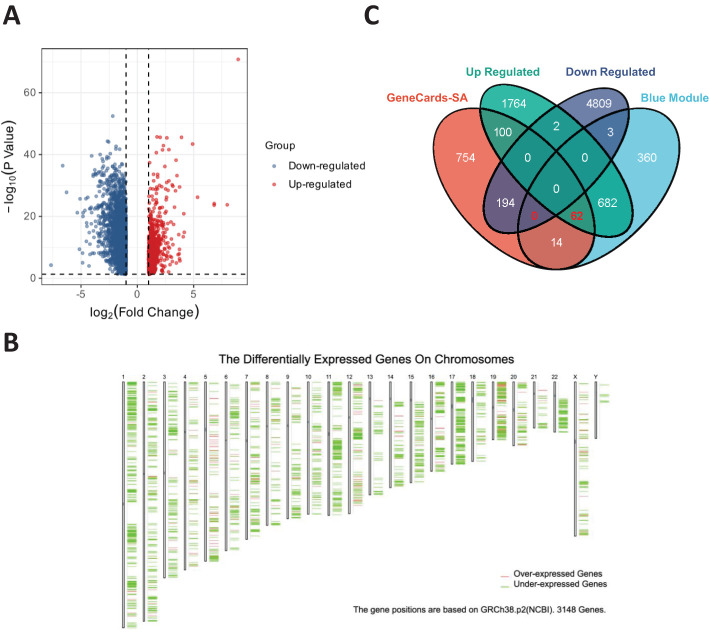
**(A)** Volcano plot of DEGs in OV. **(B)** Chromosomal positions of DEGs in OV. **(C)** Intersecting genes among DEGs in OV, blue module genes, and SA-related genes from GeneCards.

**Figure 3:**
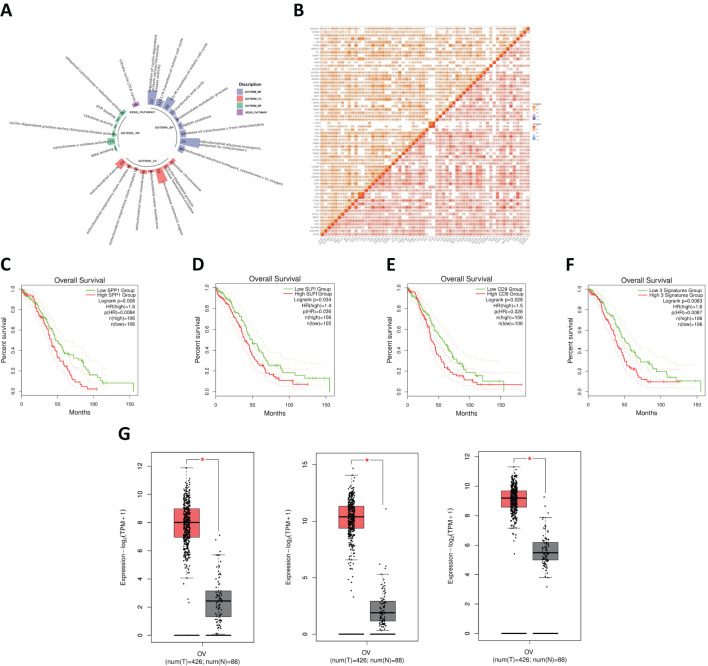
**(A)** Functional enrichment analysis of SA-related DEGs in OV. **(B)** Correlation analysis of SA-related DEGs in OV. **(C)** Survival analysis for SPP1. **(D)** Survival analysis for SLPI. **(E)** Survival analysis for CD9^+^ T cells. **(F)** Combined survival analysis of the SPP1–SLPI–CD9 gene set. **(G)** Comparison of the expression levels of key genes between normal and OV samples.

A volcano plot of the DEGs in OV obtained using the limma R package is shown in **Figure 2A**. After identifying the DEGs, we analyzed their chromosomal positions (**Figure 2B**). We further selected the genes by intersecting OV DEGs, blue module genes, and SA-related genes from GeneCards and identified 62 SA-related OV DEGs (**Figure 2C**). These 62 genes were subjected to GO and KEGG enrichment analyses. The results of the GO enrichment analysis indicated that these genes were enriched mainly in mitochondrial electron transport, ubiquinol to cytochrome c, telomeric regions, and cytochrome c oxidase activity. The KEGG enrichment analysis revealed that these genes were involved primarily in the TCA cycle (**Figure 3A**). **Figure 3B** displays the correlations among SA-related DEGs in OV.

### 3.3 Hub gene selection

#### 3.3.1 Survival analysis

We conducted a survival analysis of the 62 common genes mentioned earlier, and the data indicated that the survival outcomes associated with the genes SPP1, SLPI, and CD9 were relatively poor (**Figures 3C, D**). OV patients with high levels of SPP1, SLPI, and CD9 had significantly shorter survival times. We subsequently performed a joint survival analysis of the SPP1–SLPI–CD9 three-gene set (**Figure 3E**). Patients with high expression levels of these three genes experienced significantly shorter overall survival than patients with low expression levels (HR = 1.6, p = 0.0087, n = 106). Therefore, considering the results of the survival analysis, we identified SPP1, SLPI, and CD9 as three genes that may play important roles in the prognosis of OV.

#### 3.3.2 Comparison of the expression levels of key genes between OV samples and normal samples

The three genes screened in the previous section were compared. The results revealed that the expression levels of these three genes were significantly higher in OV samples than in normal samples (**Figure 3G**).

#### 3.3.3 Distribution of hub genes in OV immune subtypes and OV molecular subtypes

**Figure 4:**
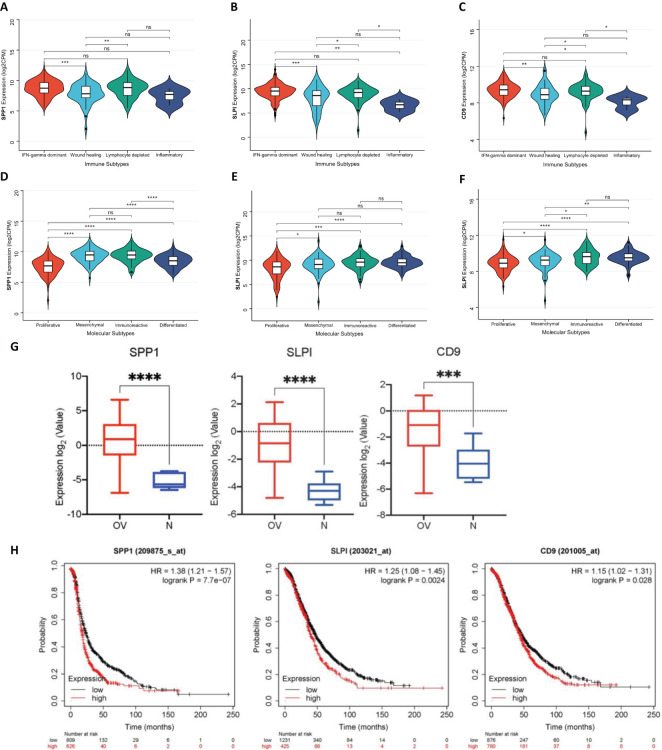
**(A)** Distribution of SPP1 expression in different OV immune subtypes. **(B)** Distribution of SLPI expression in different OV immune subtypes. **(C)** Distribution of CD9 expression in different OV immune subtypes. **(D)** Distribution of SPP1 expression in different OV molecular subtypes. **(E)** Distribution of SLPI expression in different OV molecular subtypes. **(F)** Distribution of CD9 expression in different OV molecular subtypes. **(G)** Expression levels of SPP1, SLPI, and CD9 across different groups. **(H)** Survival analysis of patients using the Kaplan-Meier plotter database. (*: P < 0.05, The results are significant at the 0.05 level; **: P < 0.01, The result is significant at the 0.01 level; ***: P < 0.001, The result is significant at the 0.001 level; ****: P < 0.0001).

We systematically observed the distribution of the expression of hub genes in the immune subtypes and molecular subtypes of OV to explore the relationships between the immune characteristics of OV and tumor subtypes (**Figure 4**). First, we conducted detailed observations of the distributions of the expression of the SPP1, SLPI, and CD9 genes in the immune subtypes of OV (**Figures 4A, B, C**). The results revealed the potential immunoregulatory roles of the hub genes in different OV subtypes. We then extensively observed the distributions of the expression of the SPP1, SLPI, and CD9 genes in the molecular subtypes of OV (**Figures 4D, E, F**). These molecular subtypes include the proliferative, mesenchymal, immunoreactive, and differentiated subtypes. The significant differences between different subtypes suggest that hub genes play distinct biological roles in different molecular subtypes of OV. The diversity of hub genes involved in the pathobiology of OV, as well as their various contributions to tumor development and the treatment response in each subtype, are highlighted.

### 3.4 Verification of hub genes

Using the GSE40595 dataset from the GEO database, the expression of SPP1, SLPI, and CD9 was analyzed (**Figure 4G**). The results clearly showed a significant difference in the expression levels of these three key hub genes between OV tissues and normal tissues. Furthermore, we validated the survival analysis of patients stratified by SPP1, SLPI, and CD9 expression using the Kaplan-Meier plotter database (**Figure 4H**). The prognosis was worse for the group with high expression of the hub genes.

### 3.5 Differential expression of the hub genes across cancers

**Figure 5:**
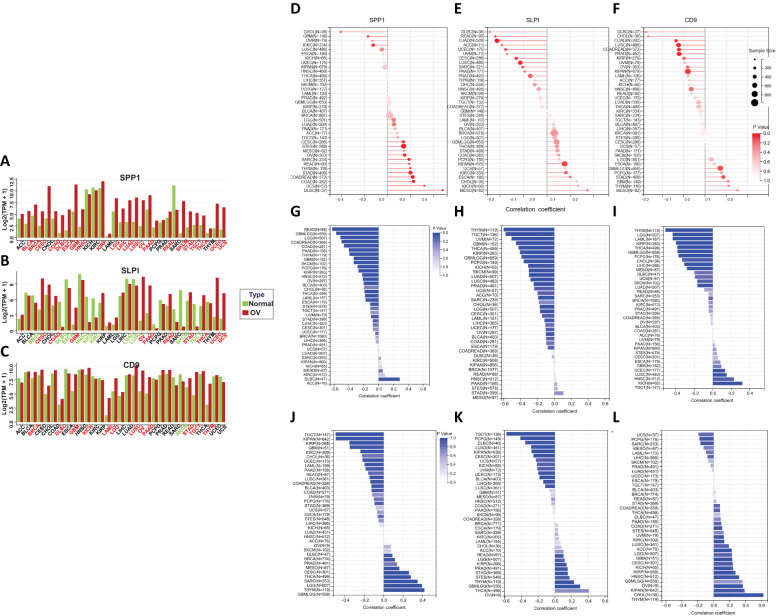
**(A–C)** Differential expression of hub genes across various cancers. **(D–F)** The correlation between the tumor mutational burden (TMB) and the expression of hub genes. **(G–I)** The correlation between RNA stemness (RNAs) and hub gene expression. **(J–L)** The correlation between DNA stemness (DNAss) and hub gene expression.

**Figure 6:**
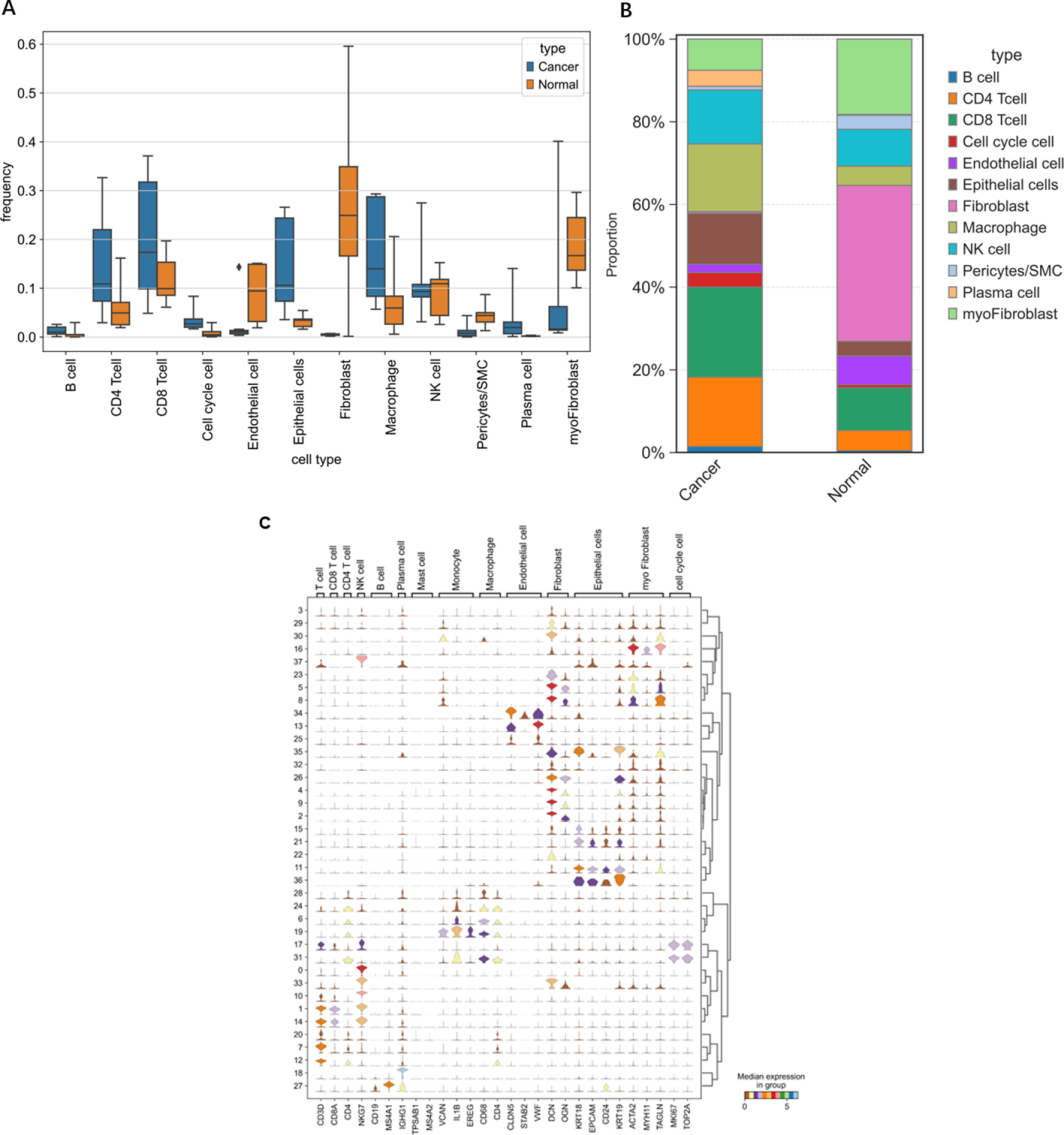
Differences in various cell types between OV individuals and normal individuals. **(A)** Frequency of occurrence. **(B)** Proportion of cells grouped based on differences in the average expression levels within groups. **(C)** Violin plots of selected marker genes for multiple cell subpopulations.

We conducted a comprehensive evaluation of the expression of the hub genes in a diverse range of cancer samples covered by The Cancer Genome Atlas (TCGA) (**Figures 5A, B, C**). The analysis revealed that high levels of SPP1 expression were widespread across the 22 cancer types studied. High SLPI expression was detected in 10 cancer types, and low SLPI expression was detected in 10 cancer types. High CD9 expression was detected in 12 cancer types, and significantly low CD9 expression was detected in only 1 cancer type. We further explored the correlation between tumor mutation burden (TMB) and hub gene expression (**Figures 5D, E, F**), the relationship between tumor stemness-related RNA scores and hub gene expression (**Figures 5G, H, I**), and the association between tumor stemness-related RNA scores and hub gene expression to validate the aforementioned observations (**Figures 5J, K, L**). **Figure 6A** shows that the number of tumor types with high expression of the hub genes exhibited significant positive correlations in all three validation analyses (**Figures 6B, C**).

### 3.6 ROC diagnosis

**Figure 7:**
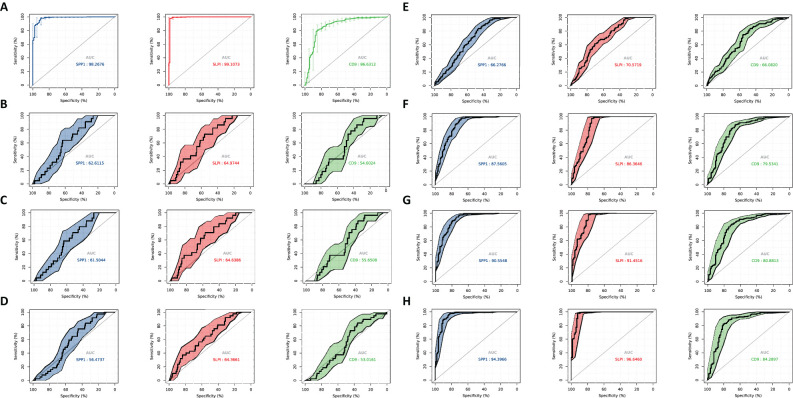
ROC curve analysis. Diagnosis of OV and time-dependent ROC at different time periods to analyze the characteristics between gene expression and mortality rate. **(A)** Diagnostic ROC for hub gene identification ROC. **(B)** 0.3 year time-dependent ROC for feature analysis between gene expression and mortality rate. **(C)** 0.5 year time-dependent ROC for feature analysis between gene expression and mortality rate. **(D)** 1-year time-dependent ROC for feature analysis between gene expression and mortality rate. **(E)** 3-year time-dependent ROC for feature analysis between gene expression and mortality rate. **(F)** 5-year dependence on ROC for feature analysis between gene expression and mortality rate. **(G)** 7-year time-dependent ROC for feature analysis between gene expression and mortality rate. **(H)** 9-year time-dependent ROC for feature analysis between gene expression and mortality rate.

We evaluated the performance of different diagnostic tests by constructing ROC curves and calculating the AUCs. We performed a time-dependent ROC curve analysis for the OV diagnosis and for different periods to analyze the relationships between gene expression levels and mortality rates (**Figure 7**). Over time, the AUC of the hub genes approached 1, indicating that the diagnostic accuracy increased over time, and the correlation between hub gene expression and mortality rates became stronger over time.

### 3.7 Nomograms and prognostic analysis

**Figure 8:**
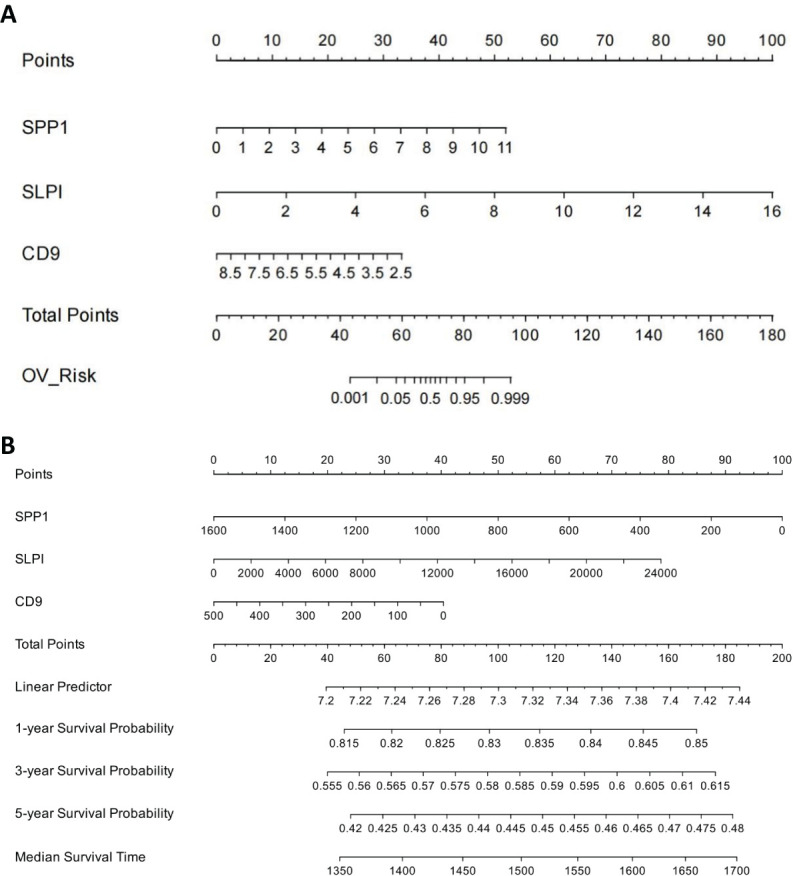
Construction of Nomogram Logistic Regression Model for Core Genes. **(A)** Nomogram-logistic regression model. **(B)** Nomogram–Cox regression model.

**Figure 9:**
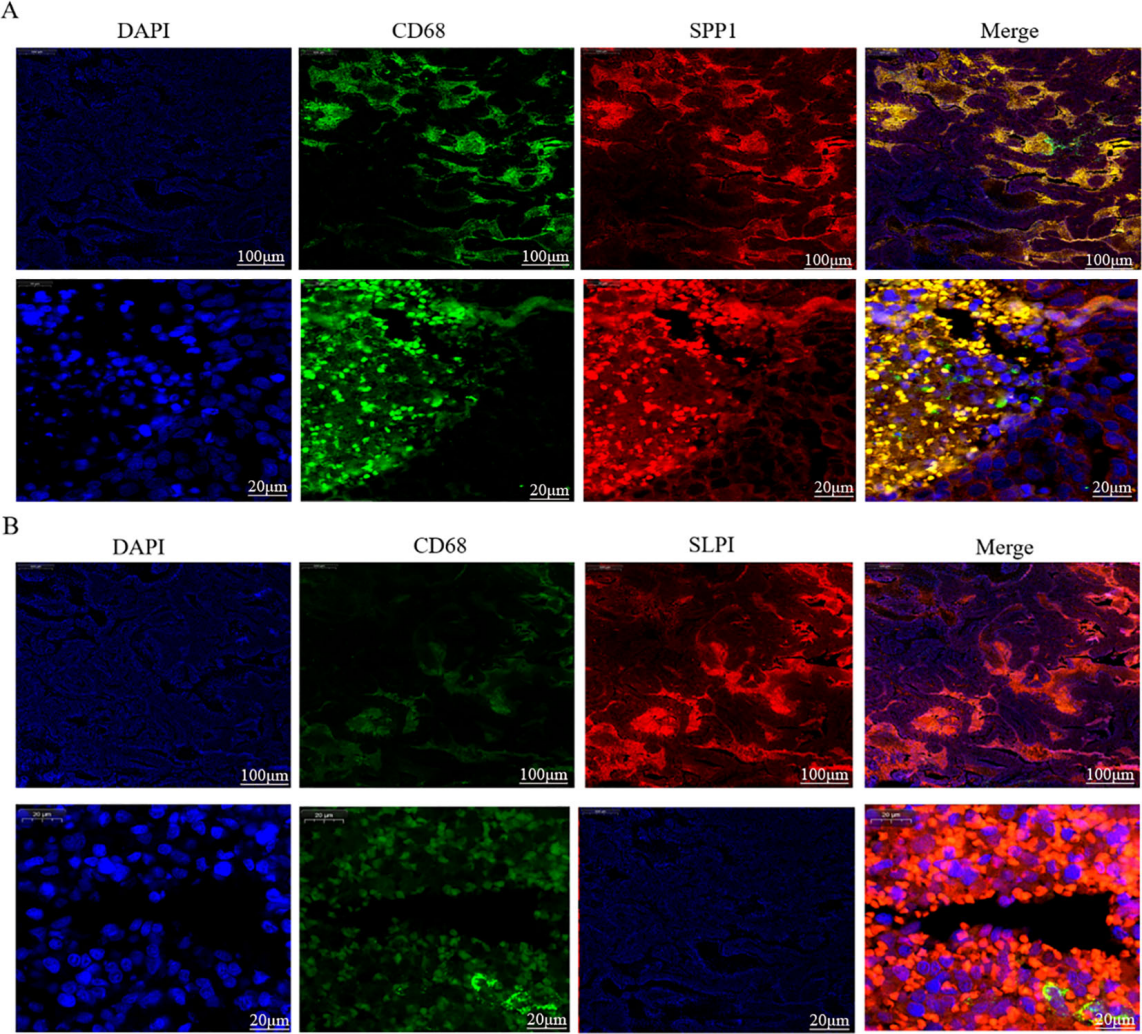
Dual immunofluorescence staining and localization of the hub proteins in OV tissues. **(A)** Colocalization of SPP1 according to CD68 immunofluorescence double staining. **(B)** Colocalization of SLPI according to CD68 immunofluorescence double staining.

**Figure 10:**
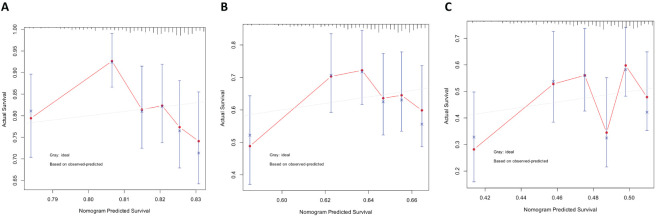
Predicting patient survival rates at 1, 3, and 5 years using nomograms. **(A)** Calibration curve of the nomogram for predicting the 1-year survival rate. **(B)** Calibration curve of the nomogram for predicting the 3-year survival rate. **(C)** Calibration curve of the nomogram for predicting the 5-year survival rate.

We established a line chart model including important predictive factors in logistic regression and Cox analyses to predict the prognosis of patients with OV (**Figure 8**). The nomogram–logistic regression model is shown in **Figure 9**. For example, if all three hub genes are expressed, with an SPP1 expression level of 6 and a score of 28 points, an SLPI expression level of 6 and a score of 38 points, and a CD9 expression level of 5 and a score of 20 points, the total score is 86 points. This score indicates a greater than 95% probability of having OV. The nomogram–Cox regression model, as shown in **Figure 9**, predicts that when all three hub genes are expressed, with an SPP1 expression level of 800 points, an SLPI expression level of 12000 points, a score of 40 points, and a CD9 expression level of 100 points, the total score is 124 points. The patient survival rate is approximately 83.7% at 1 year, 59.3% at 3 years, and 45.4% at 5 years. The prediction of the 1-, 3-, and 5-year survival rates using the nomogram indicated that patient survival rates gradually decreased over time (**Figure 10**).

### 3.8 scRNA-seq

Through single-cell sequencing, we detected significant differences in the frequencies of various cell types between OV patients and normal individuals (**Figure 6**). Specifically, in OV patients, the frequencies of B cells, CD4^+^ cells, CD8^+^ cells, cells in the cell cycle, epithelial cells, macrophages, and plasma cells were significantly increased, and the frequencies of endothelial cells, NK cells, and pericytes/SMCs did not noticeably increase. Notably, since our normal samples were obtained from the ovarian tissue of elderly individuals, which exhibited a certain degree of fibrosis, fibroblasts and myofibroblasts were more frequently present. After considering this characteristic, we chose not to consider these two cell types further in our analysis.

**Figure 11:**
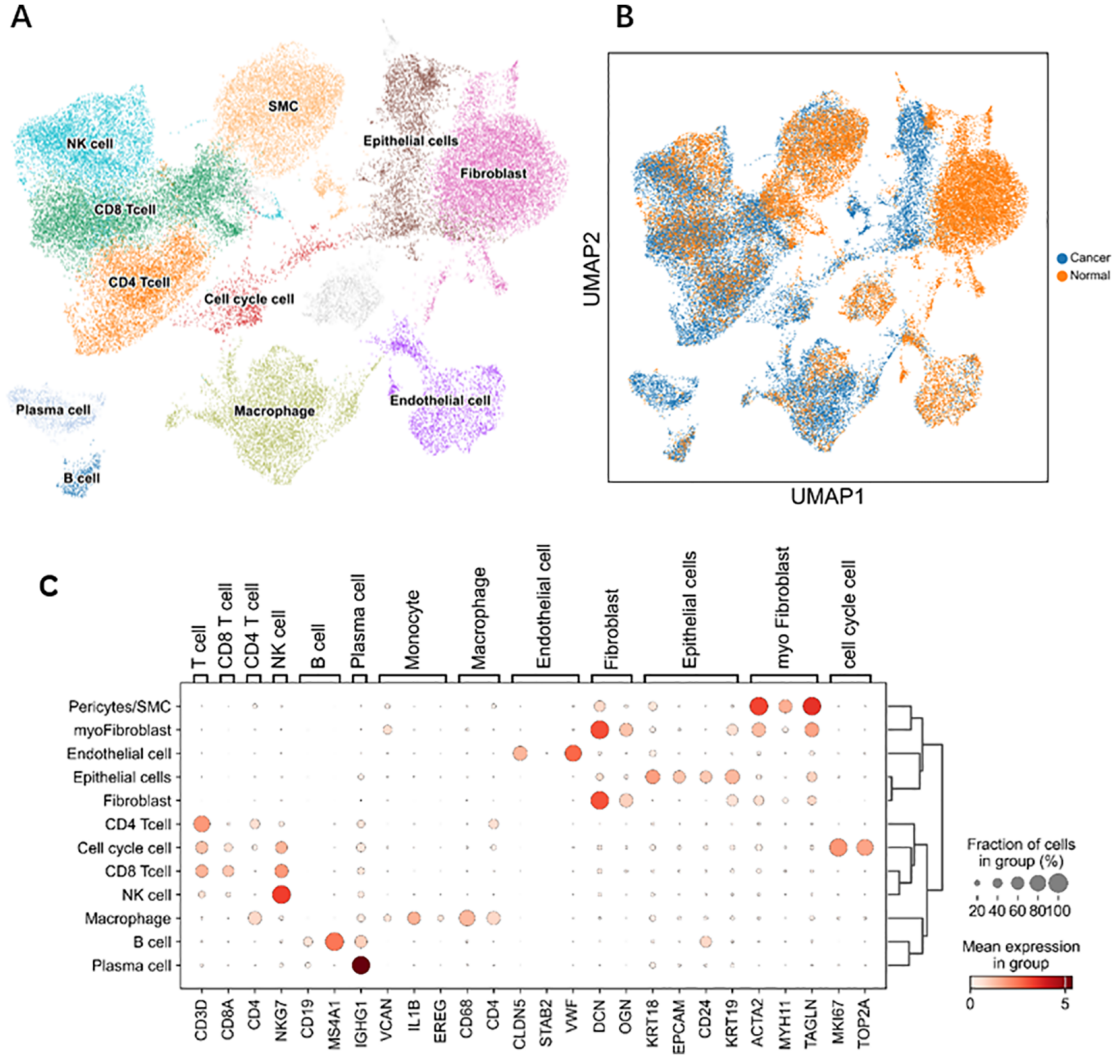
Single-cell RNA Sequencing. **(A)** Cells color-coded by cluster. **(B)** Cells color-coded according to their origin. **(C)** Dot plots of the genes expressed in each cell type.

**Figure 12:**
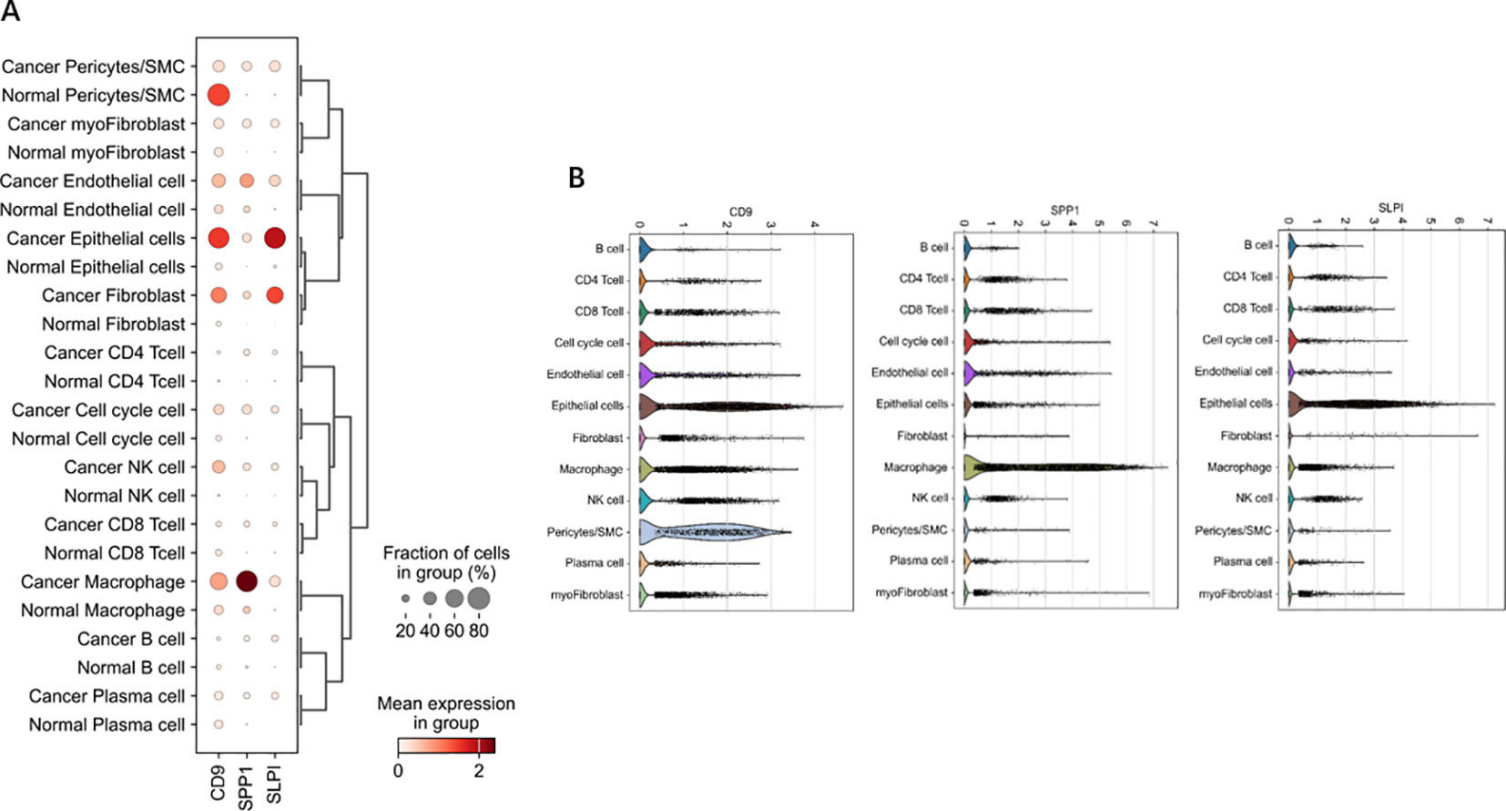
Dot and violin plots of hub genes expressed in different cell types. **(A)** The expression patterns of genes (including central genes) in diferent cell types were demonstrated through bubble plots. **(B)** The violin plot illustrates the expression patterns of genes (including central genes) in different cell types.

In **Figures 11**, **12**, the dot size indicates the proportion of cells expressing the given gene in each cell type, and the intensity of the color indicates the expression level in the expressing cells based on quantile counts. We present dot plots and violin plots of genes, including hub genes, to display the expression patterns of different genes in various cell types. Specifically, CD9 is expressed predominantly in pericytes/SMCs and epithelial cells, SPP1 is significantly expressed in macrophages, and SLPI is enriched mainly in epithelial cells. The distinct expression patterns of these genes provide important clues about their functions and potential biological roles in different cell types.

### 3.9 Expression levels of hub proteins in OV and normal cells

**Figure 13:**
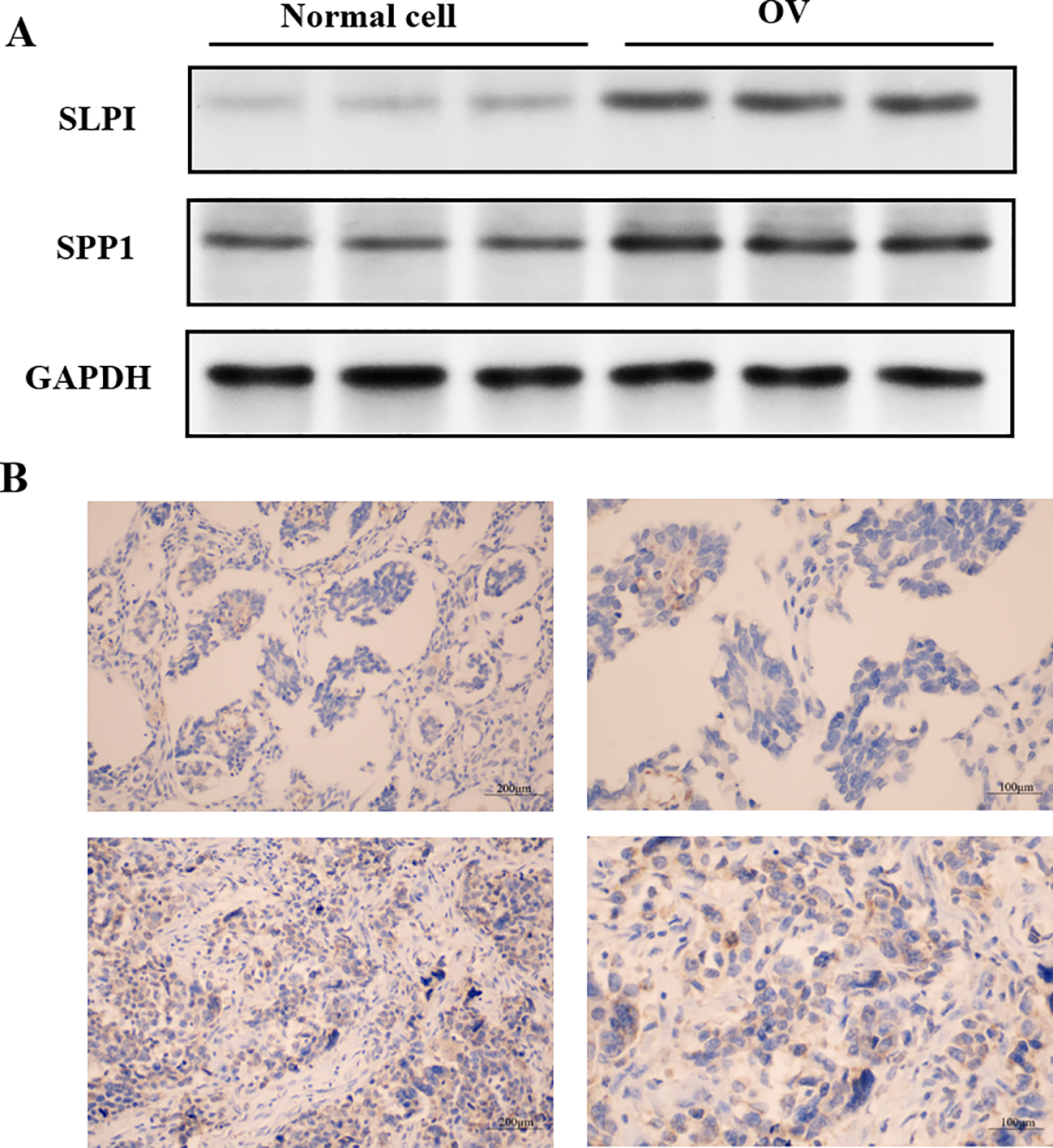
Localization of hub gene immunohistochemical staining in OV tissues. **(A)** Expression levels of the hub proteins in OV and normal cells. **(B)** Immunohistochemical staining showing the localization of the hub genes in OV tissues.

The above analyses indicate that succinic acid might act on three key genes in OV, namely, SPP1, SLPI, and CD9. CD9 expression levels are associated with the invasiveness and prognosis of tumors in cancer research. Therefore, our experiments validated the previously unreported high expression of SPP1 and SLPI in OV, as shown in **Figure 13A**.

### 3.10 Immunohistochemical staining for the hub proteins in OV tissues

The levels of hub gene-encoded protein immunopositivity were significantly increased in the OV midmembrane. As shown in **Figure 13B**, in OV tissues, the inflammatory response mainly occurred in the tumor midmembrane and outer membrane, and the inflammatory response occurred mainly through the activation of various inflammatory cells, such as T cells, B cells, macrophages, and neutrophils.

### 3.11 Double immunofluorescence staining and localization of the hub proteins in OV tissues

We further verified the associations between the hub genes and macrophages by performing dual immunofluorescence staining analysis to examine the colocalization of the hub proteins and the macrophage surface marker protein CD68 in the OV mesothelium (**Figure 9**). The results initially revealed the significant infiltration of macrophages into the OV mesothelium. Furthermore, in the OV outer membrane, the hub genes and CD68 were often coexpressed in the same cells, suggesting that further research into the role of hub genes in regulating macrophage functions has potential significance.

## 4 Discussion

This study investigated three key genes in ovarian cancer (OV), namely, SPP1, SLPI, and CD9. Studies have examined these genes in OV, and SPP1 plays various biological roles in the human body. In the skeletal system, it is involved in bone formation and regeneration processes. SPP1 is critical for skeletal development, mineralization, and bone remodeling (31). It acts as an extracellular matrix protein, influencing cell adhesion, migration, and signaling by binding to specific receptors on the cell surface (32, 33).

Additionally, SPP1 plays a vital regulatory role in the immune system (34, 35). It is involved in inflammatory responses and the activation of immune cells, recruiting immune cells to inflammatory sites, enhancing inflammatory responses, and regulating the activity of immune cells. SPP1 is also closely associated with tumor development and metastasis. It has been extensively studied in tumors because it regulates tumor cell proliferation, migration, invasion, and metastatic capabilities. High levels of SPP1 expression are correlated with tumor aggressiveness and a poor prognosis. Overall, SPP1 is an important extracellular matrix protein involved in biological processes such as skeletal development, immune regulation, and tumor progression. Research on the functions and mechanisms of action of SPP1 in related diseases could provide new targets and methods for future treatment strategies. Secretory leukocyte protease inhibitor (SLPI) is a protein produced by various cells and is widely distributed in different tissues and body fluids (36). It acts as a natural inhibitor of proteases belonging to the protease inhibitor family. SLPI performs multiple biological functions in the human body, the most important of which are related to the natural immune system. CD9 has also been shown to participate in inflammation and immune responses (37, 38).

An analysis of the distributions of hub gene expression in OV immune subtypes and OV molecular subtypes revealed that the expression of the hub genes differed significantly (39). Specifically, significant differences existed between the proliferative subtype and the mesenchymal, immunoreactive, and differentiated subtypes; between the mesenchymal subtype and the differentiated subtype; and between the immunoreactive and differentiated subtypes (21). These results suggest that the hub genes play different biological roles in various OV molecular subtypes. The significant differences in hub expression levels across subtypes may reflect the diverse roles of these genes in the pathological biology of OV and their different contributions to tumor development and treatment responses across subtypes. The single-cell sequencing analysis revealed that CD9 was highly expressed primarily in pericytes/SMCs and epithelial cells, SPP1 was significantly expressed in macrophages, and SLPI was enriched mainly in epithelial cells. Hub genes, which play important roles in inflammatory responses and immune reactions, may be involved in the infiltration of immune cells. When body tissues are damaged or infected, immune cells migrate from the blood through the vessel wall to the damaged tissue, combating and eliminating potential pathogens or abnormal cells through signal transduction and chemotactic factors. Studies have shown that the density of immune cell infiltration in OV tumors is significantly lower than that in noncancerous tissues (40). This reduction in density could result from cancer cells inhibiting the chemotaxis and infiltration of immune cells. According to our immunohistochemistry results, the hub genes were significantly positively expressed in the membranes of OVs, and inflammatory responses mostly occurred in the tumor mesothelium and outer membrane, primarily through the activation of various inflammatory cells, such as T cells, B cells, macrophages, and neutrophils. Subsequent experiments using dual immunofluorescence staining confirmed the colocalization of hub proteins and the macrophage surface marker protein CD68 in the OV mesothelium. These results first indicated significant macrophage infiltration in the OV mesothelium (37, 41).

Furthermore, the hub genes and CD68 are often coexpressed in the same cells in the OV outer membrane, suggesting that further research into the role of the hub genes in regulating macrophage functions is potentially important (15). This study thoroughly explored the potential therapeutic targets and mechanisms of succinic acid and OV, as well as differences in immune cell infiltration and gene expression patterns, providing important clues for future tumor immunotherapy research and revealing new directions for pathological biology research on OV (32). These results provide potential new strategies for diagnosing and treating tumors, with the aim of improving the patient prognosis and survival rates (38). The study comprehensively investigates the roles of SPP1, SLPI, and CD9 in ovarian cancer, providing insights into their functions in skeletal development, immune regulation, and tumor progression. It highlights the significant differences in gene expression across molecular and immune subtypes, revealing their diverse contributions to tumor biology. The study also explores the co-localization of hub genes with immune cells, suggesting their involvement in immune cell infiltration. These findings offer valuable clues for developing new diagnostic and therapeutic strategies for ovarian cancer. While the study provides valuable insights, it may lack in-depth functional validation through *in vivo* experiments. The mechanisms by which these genes regulate immune cell infiltration and tumor progression are not fully elucidated. Additionally, the study’s focus on gene expression and co-localization may not fully capture the dynamic interactions between hub genes and the tumor microenvironment. Further research is needed to explore the detailed pathways and potential therapeutic applications of these findings (42).

## 5 Conclusion

This study investigated the potential therapeutic targets and mechanisms of succinic acid in ovarian cancer and the differences in immune cell infiltration and gene expression patterns, providing important insights for future tumor immunotherapy research. These findings provide new directions for pathological biology research on ovarian cancer and offer potential new strategies for diagnosing and treating tumors to improve the patient prognosis and survival rates.

## Data Availability

The datasets presented in this study can be found in online repositories. The names of the repository/repositories and accession number(s) can be found below: https://www.genecards.org, GEO.
